# Impact of Previous Continuous Positive Airway Pressure Use on Noninvasive Ventilation Adherence and Quality in Obesity Hypoventilation Syndrome: A Pragmatic Single-Center Cross-Sectional Study in Martinique

**DOI:** 10.3390/biomedicines11102753

**Published:** 2023-10-11

**Authors:** Moustapha Agossou, Bérénice Awanou, Jocelyn Inamo, Mickael Rejaudry-Lacavalerie, Jean-Michel Arnal, Moustapha Dramé

**Affiliations:** 1 Department of Respiratory Medicine, CHU of Martinique, 97261 Fort-de-France, France; sessito.awanou@chu-martinique.fr; 2 Department of Cardiology, CHU of Martinique, 97261 Fort-de-France, France; jocelyn.inamo@chu-martinique.fr; 3 Department of Neurophysiology, CHU of Martinique, 97261 Fort-de-France, France; mickael.lacavalerie@chu-martinique.fr; 4 Intensive Care Unit, Hôpital Sainte Musse, 83100 Toulon, France; jean-michel@arnal.org; 5 Department of Clinical Research and Innovation, CHU of Martinique, 97261 Fort-de-France, France; moustapha.drame@chu-martinique.fr; 6 EpiCliV Research Unit, Faculty of Medicine, University of the French West Indies, 97261 Fort-de-France, France

**Keywords:** obesity hypoventilation syndrome, obstructive sleep apnea, CPAP, noninvasive ventilation, NIV adherence

## Abstract

There is a strong relationship between obstructive sleep apnea (OSA) and obesity hypoventilation syndrome (OHS). When OHS is combined with severe OSA, treatment consists of continuous positive airway pressure (CPAP), followed by noninvasive ventilation (NIV) in the case of CPAP failure. Currently, the impact of a previous use of CPAP on the quality of NIV is unknown. We conducted a cross-sectional study with OHS patients, to assess the quality of NIV according to previous CPAP use. We included 75 patients with OHS on NIV (65 women, 87%). Among these, 40 patients (53.3%) who had had prior CPAP (CPAP+ group) were compared to the remaining 35 patients (46.7%) (CPAP− group). Key characteristics were comparable between the CPAP+ and the CPAP− groups: age at diagnosis of OHS was 67 ± 3 vs. 66 ± 4 years (*p* = 0.8), age at inclusion was 73 ± 15 vs. 69 ± 15 years (*p* = 0.29), number of comorbidities was 3.7 ± 1.2 vs. 3.3 ± 1.5, the Charlson index was 5.1 ± 2 vs. 4.6 ± 1.8, and BMI was 41.6 ± 7.6 kg/m^2^ vs. 41.2 ± 8.2, respectively, all *p* > 0.05. Follow-up length was greater in CPAP+ vs. CPAP− patients (5.6 ± 4.2 vs. 2.9 ± 2.9 years, *p* = 0.001). The quality of NIV based on daily adherence, pressure support, apnea–hypopnea index (AHI) and leaks was similar in both groups. Reduced adherence (less than 4 h daily) was found in 10 CPAP+ patients (25%) versus 7 CPAP− patients (20%), *p* = 0.80. NIV efficacy was also similar. This study found no difference in the quality of NIV or in adherence between patients who had had prior CPAP and those who had not. Previous CPAP does not appear to improve the quality of NIV.

## 1. Introduction

The worldwide prevalence of obesity is increasing. It is estimated by the World Health Organization (WHO) that approximately 4 million deaths each year are attributable to obesity globally [1]. Obesity hypoventilation syndrome (OHS) is a chronic respiratory failure that is related to obesity. It is defined as the simultaneous presence of obesity (namely, body mass index (BMI) of ≥30 kg/m^2^) and daytime hypercapnia (pCO_2_ > 45 mmHg) in the absence of other causes of alveolar hypoventilation [2]. Obstructive sleep apnea is frequently associated with OHS [2]. OHS carries substantial morbidity and mortality [2], with high consumption of healthcare in affected individuals [3,4]. The treatment of OHS has evolved over time and has long relied on noninvasive ventilation (NIV). Recent research has highlighted the role of continuous positive airway pressure (CPAP) therapy in the management of OHS in stable patients, notably when diagnosed in a sleep laboratory [5,6,7,8]. Accordingly, the latest recommendations from the American Thoracic society (ATS) proposed the introduction of first-line CPAP treatment in stable OHS, with a switch to NIV in the event of CPAP failure [9]. While NIV can be an effective treatment for OHS, its success depends on patient adherence, which improves efficacy and arterial blood gases, quality of life, and mortality [10]. Efficiency of the treatment also depends on the quality of the NIV in terms of leaks or obstructive events [11].

The clinician’s task is to find strategies that will encourage the patient to adhere to a treatment regimen that may necessitate changes to their lifestyle. Thus, there is value in seeking all the possible levers that could be used to improve NIV compliance. Ennis et al. identified several factors that were associated with improved adherence to NIV in patients with nocturnal hypoventilation, including previous experiences with positive airway pressure therapy, subjective symptom improvement, familiarity with medical treatments, understanding of nocturnal hypoventilation and its consequences, support from family and healthcare providers, and early adaptation to treatments [12]. CPAP treatment uses similar equipment to NIV, and patients may thus feel familiar with the equipment and treatment. Because it also uses pressure to keep the airway open, one might imagine that CPAP creates a ventilation memory in the patient. In any case, there is little change in the patient’s environment during the transition from CPAP to NIV. In daily practice, it is common to encounter patients already receiving CPAP for OSA, who then present hypercapnic respiratory insufficiency leading to a diagnosis of OHS. In this context, these patients are switched from CPAP to NIV.

We hypothesized that previous CPAP use would enhance the adherence to home nocturnal NIV in patients with OHS. Therefore, the aim of this study was to compare the adherence and quality of NIV in OHS patients who had previously received a CPAP device for OSA with those who had not. The primary outcome measure was NIV adherence, and secondary outcomes included NIV quality, assessed by leaks and the apnea–hypopnea index (AHI), and NIV efficacy, assessed by daytime partial pressure of carbon dioxide (pCO_2_).

## 2. Patients and Methods

We performed an observational, cross-sectional, single-center study in a cohort of OHS patients from the Department of Respiratory Medicine at the University Hospital (CHU) of Martinique. All patients followed-up for OHS in our department are systematically entered into a database. The Respiratory Medicine Department is responsible for the management and care of all patients with chronic respiratory diseases on our island. Patients were adults (>18 years) diagnosed with OHS based on body mass index (BMI) > 30 kg/m^2^, daytime hypercapnia, and the absence of another cause of hypoventilation. We excluded patients with a smoking history > 10 pack-years in women or 15 pack-years in men and patients associated with an obstructive pattern in spirometry (FEV1/FVC < 70%) in whom associated chronic obstructive pulmonary disease (COPD) cannot be ruled out [13,14].

At the time when OHS was diagnosed, patients systematically underwent screening for OSA (polygraphy or polysomnography, unless they already had documented and treated OSA) and (as far as possible) a pulmonary functional test and echocardiography. If pulmonary hypertension was suspected, exploratory right heart catheterization was performed to confirm or rule out the diagnosis. Polygraphy/polysomnography data were interpreted according to the International Classification of Sleep Diseases (ICSD-3 2023). Severity was defined based on AHI and classified as follows: absent if AHI < 5/h, mild OSA with AHI of 5 to <15, moderate with AHI of 15 to 29, and severe with AHI of ≥30. In the case of hypercapnic acute respiratory insufficiency, NIV was initiated during hospitalization. Settings were fixed arbitrarily at the outset and adjusted during hospitalization. For patients who had previously been receiving CPAP, PAP was determined by subtracting 2 cmH_2_O from the average pressure 90% of the time. For other patients, PAP was determined at the physician’s discretion and then adjusted on the basis of the residual AHI. Support pressure was determined with a target tidal volume of 8 mL/kg of theoretical bodyweight calculated according to the Lorentz formula and optimized based on response to CO_2_ and leaks. For initiation of CPAP, we started with three days of auto-adjusting CPAP and then set the device in fixed mode to the average pressure 90% of the time. Of note, patients who had previously received CPAP for OSA all had auto-adjusting devices, and they did not undergo screening for OHS.

All patients had regular follow-up in outpatient consultations, and at each follow-up, we recorded mean adherence for the last 90 days, blood gases, ventilatory parameters, and data reflecting the quality of NIV (leaks and AHI). Patients included in the study were seen in consultation between 1 January 2022 and 31 October 2022. We collected sociodemographic data (age, age at diagnosis, sex, BMI), history of previous use of PAP devices for OSA at time of diagnosis, arterial blood gas at diagnosis, last blood gas in a stable state, and spirometry data at inclusion. Daily adherence, leaks, and AHI were measured for the last 90 days, and NIV parameters (IPAP, EPAP, and pressure support) were measured at inclusion, using stored data from the devices.

Functional tests were interpreted according to the EU Standard criteria, taking ethnicity into account [15,16,17].

The study was performed in accordance with the Declaration of Helsinki and received the approval of the Institutional Review Board of the University Hospitals of Martinique (under the number 2022/158).

We performed descriptive analysis. We describe quantitative variables as mean ± standard deviation (SD) and qualitative (categorical) variables as count and percentage. Continuous variables were compared using Student’s *t*-test and proportions with the chi square or Fisher’s exact test as appropriate. Normality of distributions was tested using the Shapiro–Wilk test. Bivariable analyses for the association between previous use of CPAP and daily adherence, quality of NIV, and efficacy were performed using binary logistic regression modeling. Statistical analyses were performed using SAS software version 9.4 (SAS Institute Inc., Cary, NC, USA). *p*-values < 0.05 were considered statistically significant.

## 3. Results

We included 75 patients with OHS using NIV, 65 (87%) of whom were women. Of the entire group, 40 (53%) had used CPAP previously (CPAP+ group), with the remaining 35 (47%) having gone directly onto NIV (CPAP− group). Key characteristics were comparable in the CPAP+ versus CPAP− groups as follows: mean age at diagnosis of OHS was 67 ± 3 vs. 66 ± 4 years, mean age at inclusion was 73 ± 15 versus 69 ± 15 years, mean number of comorbidities was 3.7 ± 1.2 versus 3.3 ± 1.5 (*p* = 0.3), mean Charlson index was 5.1 ± 2 versus 4.6 ± 1.8 (*p* = 0.3), and mean BMI was 41.6 ± 7.6 kg/m^2^ versus 41.2 ± 8.2 (*p* = 0.8), respectively. The average length of follow-up was 5.6 ± 4.2 years in the CPAP+ group versus 2.9 ± 2.9 years in the CPAP− group (*p* = 0.001).

The comorbidities and therapeutic characteristics of the patients are detailed in Table 1.

The spirometric data at inclusion are displayed in Table 1.

Adherence to NIV and quality of ventilation including ventilator settings, leaks, and residual AHI were similar in the two groups (Table 2).

Ten patients (25%) in the CPAP+ group and seven (20%) in the CPAP− group used NIV less than a mean of 4 h daily over 90 days (*p* = 0.8). There were also no differences in NIV efficacy between the two groups based on hypercapnia resolution (*p* = 0.3).

## 4. Discussion

In this study, we found that there was no significant difference in adherence to NIV between those individuals who were previously managed with CPAP and those who were prescribed NIV as an initial therapy. Similarly, no between-group differences in the quality of ventilation were reflected in residual AHI and leaks nor in the effectiveness of NIV measured by the resolution of hypercapnia among this population of patients with OHS. Because it is a pragmatic study, we were considering a calculation of power a posteriori. Because the averages are strictly the same (6.2 ± 3.2 versus 6.1 ± 3.2), this does not seem relevant.

Adherence to NIV is known to be an important predictor of treatment success in patients with hypercapnic chronic respiratory failure [10], and previous experience with therapy has been identified as a factor that can influence adherence in nocturnal hypoventilation [9]. While CPAP treatment is a viable alternative to NIV in stable OHS [6,8,9,10], it is important to note that the long-term effectiveness of CPAP may wane over time, leading to a transition to NIV in some cases [11]. In our cohort, 53% of patients had already received CPAP treatment for OSA before being diagnosed with OHS, and NIV treatment is recommended for those who continue to experience nocturnal hypoventilation despite using CPAP [6]. Adherence to NIV and correction of hypercapnia are critical to the effective management of nocturnal hypoventilation. A number of studies have demonstrated that persistent hypercapnia is linked to high mortality rates and significant healthcare utilization in affected individuals [1,2,18]. In this context, adherence to treatment is a key determinant of patient outcomes [7]. Molkhlesi et al. found that daily adherence to PAP of 5 to 7 h was associated with improved arterial blood gas parameters in patients with OHS [10]. Castro-Anon reported that NIV adherence of less than 4 h per day was linked to higher mortality in this patient population [19]. The quality of NIV has predominantly been studied in patients with hypercapnic chronic respiratory failure related to neuromuscular diseases. Morelot-Panzani et al. reported that the correction of leaks, management of OSA and adaptation to the patient’s degree of ventilator dependence all improve the prognosis in amyotrophic lateral sclerosis (ALS) [11]. Georges et al. also reported that survival was reduced with upper obstructive events on NIV in ALS [20].

In this study, we hypothesized that patients who had previously received CPAP treatment might have better adherence to NIV and improved treatment outcomes, due to a possible “ventilation memory” effect. We hoped that this would lead to greater efficiency in the management of OHS. Our findings indicate that previous exposure to PAP devices does not seem to affect the quality of NIV via better adherence in patients with OHS. We observed in this study that patients were generally quite adherent overall, with an average of more than 4 h per day of use. Only a very small number of patients had daily use below 4 h during the study period. This was not evaluated at the time of NIV initiation, which may have occulted any effect of prior CPAP use over time.

We believe that patients on two-level pressure ventilation should receive the same attention and psychological support to improve adherence [16], regardless of whether they have previously used a PAP device or not.

Our study raises the question of the possible under-diagnosis of OHS. Indeed, patients being followed up in sleep clinics or by physicians specialized in sleep disorders do not undergo screening for OHS, and the diagnosis may come to be made in a context of acute respiratory insufficiency. We noted that patients who were already receiving CPAP less frequently had a diagnosis made in the context of acute respiratory failure, in line with a previous report [21]. There is clearly a need to implement systematic screening for OSA in persons with obesity, and a need of screening for OHS in those with OSA and obesity, with a view to initiating early management.

Furthermore, the patients in our study presented a predominantly restrictive syndrome, likely related to obesity. Restrictive syndrome may contribute to the occurrence of alveolar hypoventilation in obese patients. In a previous work, we already reported that the persistence of hypercapnia under NIV was more strongly associated with the severity of a restrictive syndrome than with the severity of obesity [22].

## 5. Study Strengths and Limitations

A strength of our study is that it presents data from a population of mainly Afro-Caribbean descent; a population that is markedly affected by both OSAS and obesity, but in whom OHS has not been widely investigated. This is also a population that is representative of real-life practice. The study question also has important public health implications, because CPAP is now recommended in first intention in OHS, with NIV used in case of CPAP failure, meaning that increasing numbers of patients may switch from CPAP to NIV in the future. It seems important to anticipate patient needs in terms of therapeutic education. There are a number of limitations of this study that need to be acknowledged. Firstly, it is a single-center, observational, retrospective study, which has the typical limitations associated with this design, such as the lack of knowledge about potential confounding biases, limited data availability, and a small number of patients. Additionally, we were unable to determine adherence to CPAP treatment before transitioning to NIV. Some patients had several years of NIV use, which may have allowed them time to adjust to their treatment. Due to the delay between diagnosis and study measurements, we cannot exclude the possibility that less compliant patients were no longer being followed up for various reasons (NIV discontinuation, death) and therefore could not be included in this study. Finally, we did not dispose of data regarding the initial polygraphy, which would have allowed us to compare polygraphy data between groups. The majority of patients were followed for OSA by specialists in respiratory medicine. We considered that their diagnosis, which had been made several years before, was reliable, and therefore, we did not re-conduct the examinations in view of the following factors:

- Difficulties and delays in accessing the required examinations.
- Most patients presented with acute respiratory insufficiency requiring immediate NIV or intubation.

Our results should be confirmed in prospective studies to better identify the factors that influence compliance in patients with OHS.

## 6. Conclusions 

In current medical practice, it is common for patients with OHS to switch between continuous positive airway pressure (CPAP) and noninvasive ventilation (NIV) treatment. While the quality of NIV is a crucial element in its effectiveness as a treatment, our study did not find any effect of previous CPAP use on NIV quality, via improved adherence. Based on these findings, we recommend implementing the same therapeutic education procedure for all OHS patients, regardless of whether they have previously used CPAP therapy or not.

## Figures and Tables

**Table 1:** Comorbidities, circumstances of diagnosis, and spirometry in patients undergoing noninvasive ventilation according to the previous use of continuous positive airway pressure (CPAP).

	CPAP+ N = 40	%	CPAP− N = 35	%	*p*
Age at diagnosis	67 ± 3		66 ± 4		0.8
Age at inclusion	75 ± 15		69 ± 15		0.29
Female sex	33	82.5	32	91.4	0.32
Body mass index (BMI) ≥ 40	20	36.7	19	44.4	0.6
Comorbidities					
Arterial hypertension	32	80.6	31	88.6	0.4
Diabetes mellitus	24	60	22	62.9	0.8
Asthma	17	42.5	8	22.9	0.09
Cardiac arrhythmia	5	12.5	7	20	0.5
Other heart diseases	7	17.5	7	20	1
Pulmonary hypertension	5	12.5	5	14.3	1
Lower limb arterial disease	4	10	1	2.9	0.4
Circumstances of OHS diagnosis					
Acute respiratory failure	25	52.5	31	88.6	0.02
Follow-up of sleep apnea	15	37.5	0	0	
Follow-up of another disease	0	0	1	2.9	
Unknown	0	0	3	8.6	
Pulmonary functional test at inclusion					
	CPAP+	CPAP−		*p*	
FEV1 (%) *	58.7	56.1		0.5	
FVC (%) *	63.2	58.1		0.3	
FEV1/FVC (%) **	77	77.9		0.8	
TLC (%) *	73.6	75.9		0.6	
RV (%) *	90.8	109.9		0.09	
ERV (%) *	80.3	71.4		0.6	

* % of theoretical value ** RatioX100 FEV1: forced expiratory volume in 1 s. FVC: forced vital capacity. TLC: total lung capacity. RV: residual volume. ERV: expiratory reserve volume.

**Table 2:** Adherence, quality of NIV, and arterial blood gas at inclusion in OHS patients with and without previous CPAP.

Characteristics at Inclusion	CPAP+ N = 40	CPAP− N = 35	*p*
Daily adherence (Hours)	6.2 ± 3.2	6.1 ± 3.2	0.8
Adherence < 4 h	10	7	0.6
Median unintentional leaks (L/min)	13.8 ± 23.3	13.6 ± 21.3	0.9
AHI	5.2 ± 6.6	4 ± 5.9	0.4
Hypercapnia (>45 mmHg)	27	20	0.3
EPAP (cmH_2_O)	8.7 ± 2.4	8.4 ± 2.7	0.9
IPAP (cmH_2_O)	20.6 ± 3.2	20.3 ± 2.8	0.7
Support pressure (cmH_2_O)	11.9 ± 2.6	11.9 ± 1.9	0.9
Arterial blood gas			
pH	7.38 ± 0.03	7.39 ± 0.03	0.4
pCO_2_ (mmHg)	46 ± 5	47 ± 6	0.5
pO_2_ (mmHg)	78 ± 17	71 ± 8	0.08
Bicarbonates (mmol/L)	27 ± 4	28 ± 4	0.4

CPAP: continuous positive airway pressure, AHI: apnea–hypopnea index, EPAP: expiratory positive airway pressure, IPAP: inspiratory positive airway pressure, pCO_2_: partial pressure of carbon dioxide, pO_2_: partial pressure of oxygen.

## Data Availability

The datasets generated and/or analyzed during the current study are available from the corresponding author on reasonable request.

## Abbreviations

BMI	Body mass index
CHU	University hospital center
CPAP	Continuous positive airway pressure
COPD	Chronic obstructive pulmonary disease
EPAP	Expiratory positive airway pressure
ERV	Expiratory reserve volume
FEV1	Forced expiratory volume in 1 s
FVC	Forced vital capacity
IPAP	Inspiratory positive airway pressure
mmHg	Millimeter of mercury
NIV	Noninvasive ventilation
pCO_2_	Partial pressure of carbon dioxide
pO_2_	Partial pressure of oxygen
PS	Pressure support
OHS	Obesity hypoventilation syndrome
OSA	Obstructive sleep apnea
RV	Residual volume
TLC	Total lung capacity
